# A Metabolomic Approach to Predict Breast Cancer Behavior and Chemotherapy Response

**DOI:** 10.3390/ijms19020617

**Published:** 2018-02-21

**Authors:** Marcella Regina Cardoso, Juliana Carvalho Santos, Marcelo Lima Ribeiro, Maria Cecília Ramiro Talarico, Lais Rosa Viana, Sophie Françoise Mauricette Derchain

**Affiliations:** 1 Hospital da Mulher Prof. Dr. José Aristodemo Pinotti—Centro de Atenção Integral à Saúde da Mulher (CAISM), University of Campinas (UNICAMP), Campinas, São Paulo 13083-881, Brazil; macardoso86@hotmail.com (M.R.C.); mcecilia_r@hotmail.com (M.C.R.T.); lala.viana311088@gmail.com (L.R.V.); sophie.derchain@gmail.com (S.F.M.D.); 2 Clinical Pharmacology and Gastroenterology Unit, São Francisco University, Bragança Paulista, São Paulo 13083-881, Brazil; marcelo.ribeiro@usf.edu.br

**Keywords:** breast cancer, drug resistance, metabolomics

## Abstract

Although the classification of breast carcinomas into molecular or immunohistochemical subtypes has contributed to a better categorization of women into different therapeutic regimens, breast cancer nevertheless still progresses or recurs in a remarkable number of patients. Identifying women who would benefit from chemotherapy could potentially increase treatment effectiveness, which has important implications for long-term survival. Metabolomic analyses of fluids and tissues from cancer patients improve our knowledge of the reprogramming of metabolic pathways involved in resistance to chemotherapy. This review evaluates how recent metabolomic approaches have contributed to understanding the relationship between breast cancer and the acquisition of resistance. We focus on the advantages and challenges of cancer treatment and the use of new strategies in clinical care, which helps us comprehend drug resistance and predict responses to treatment.

## 1. Introduction

Breast cancer is a worldwide public health problem in both developed and developing nations. It is the second most common cancer in women, with an estimated 1.7 million invasive breast cancer cases and 521,900 deaths in 2012 [1]. The death rate associated with breast cancer varies in different regions, depending on the diagnosis stage, treatment quality, prevalence of various subtypes, and therapy effectiveness [2,3]. Breast cancer treatments include surgery, radiation therapy, chemotherapy, hormone therapy, and targeted therapy [4,5,6].

The main obstacle that arises from the treatment of any cancer with chemotherapeutic drugs is the development of resistance. Chemoresistance enables cancer cells to survive drug attack and proliferate uncontrollably, which may lead to strong metastatic potential and disease progression [7,8,9,10,11,12]. Cancer cells can be intrinsically resistant to first-line chemotherapeutic agents or acquire resistance during treatment after long-term drug exposure [4,13].

Long-term survival rates related to breast cancer are directly correlated to early detection of disease. Thus, more sensitive biomarkers capable of detecting earlier stages of disease may contribute to the identification of molecular targets necessary for successful treatment [14]. Metabolomics has emerged as a new approach to identify and characterize biomarkers, which analyzes metabolites associated with disease from biofluids and tissues [15].

The metabolomic approach can be applied using techniques such as nuclear magnetic resonance (NMR) and mass spectrometry (MS), which offer information about a large number of metabolites through a multivariate statistical analysis. This approach allows the comparison of metabolite levels between healthy individuals and patients with diseases such as cancer [16,17]. Metabolomic analysis is used for early disease diagnosis, nutritional studies, toxicity analysis, and the evaluation of drug action, as well as studying the acquisition of resistance to chemotherapy [18]. Metabolites are final byproducts derived from the interaction between intracellular pathways and their microenvironment [19]. It has been proposed that the evaluation of a metabolite profile might allow the understanding of biochemical processes that occurred, or were occurring, at the time of breast cancer diagnosis [13,20,21]. Additionally, in the field of chemoresistance, developing sensitive prognostic tools is important to characterize the patient as an individual and to customize treatment with specific strategies aimed to maximize the drug action [22,23]. This review discusses advances in metabolomics approaches that help understand the relationship between disease and the acquisition of resistance to treatment, with a particular focus on breast cancer.

## 2. Breast Cancer Treatment According to Histological Subtype

Breast cancer is a heterogeneous disease classified into several biological, molecular, and histological subtypes that demonstrate variable prognoses and responses to chemotherapy [24]. Genetically, it can be classified into hierarchical clusters of intrinsic subtypes that have particular tumor characteristics and clinical evolution: basal, luminal A, luminal B, human epidermal growth factor receptor 2 overexpressed (HER2+), and normal [25,26]. Several commercially available tests, including prediction analysis of microarray 50 (PAM50), classify breast carcinomas into the five intrinsic subtypes [27,28]. However, other biological methods can be used for categorization, such as the reverse phase protein array based on the expression of 171 cancer-related proteins, which defines the subtypes of breast cancer as basal, HER2, luminal A, and luminal A/B. Additionally, the potentially novel protein-defined subgroups reactive I and reactive II have been identified as associated with the expression of proteins likely found in the microenvironment and/or active cancer fibroblasts around the carcinoma [29].

In clinical practice, the method for breast carcinoma classification is based on the immunohistochemical assessment of estrogen (ER), progesterone receptor (PR), and Ki67, as well as reflex fluorescence in situ hybridization of HER2 expression [30]. The luminal A subtype demonstrates strong expression of ER and PR, does not express HER2, and has low Ki67 expression, while the luminal B subtype expresses ER, high levels of Ki67, and may express PR. Tumors expressing ER and positive for HER2 are also classified into this subtype [31]. Typically, luminal subtypes have a better prognosis than non-luminal subtypes, while the luminal A subtype has a better prognosis than luminal B largely because cases of the latter have an imprecise prognosis and poor response to treatment [32]. The luminal A subtype is more common in older women who show a better response to hormone therapy and an intermediate response to chemotherapy [28,33,34]. Luminal B/HER2-positive cases have the worst prognosis and a higher incidence among young women compared with luminal B/HER2-negative cases [31,35]. The most common treatments for patients with the luminal B subtype are endocrine therapy and chemotherapy. Luminal B carcinomas have a poor response to tamoxifen because of drug resistance [36]. 

Trastuzumab, also known as humanized monoclonal antibody, is used as a treatment for luminal B/HER2-positive tumors in early and metastatic cases [37]. It interacts with HER2 and inhibits HER2/HER3 signaling and subsequent HER2 release [38]. Compared with other proteins of the HER family, there are no known mutations or alterations that result in oncogenic activity to HER3. Additionally, no transformations have been observed when HER3 is overexpressed or under continuous ligand stimulation. HER3 appears to function as a signaling substrate and specialized allosteric activation mechanism of other HER proteins [38,39]. Studies in HER2-positive breast cancer indicate that ligand-independent HER2–HER3 heterodimers behave as oncogenic inductors in trastuzumab-sensitive substrates. However, it is possible that overexpression of HER3 itself, or any of its ligands, may result in trastuzumab sensitivity [40,41]. HER2-positive patients in advanced stages who underwent trastuzumab treatment were shown to have an improved survival rate, but occasionally to experience disease progression [42]. Recently, national and international guidelines established that neoadjuvant chemotherapy should involve a combination of taxanes with a dual blockade of trastuzumab and pertuzumab in HER2-positive cases [43]. Pertuzumab acts by inhibiting HER2 dimerization with another HER (HER1–4) receptor. Its treatment choice is based on higher rates of pathological complete response (pCR) with the addition of HER2-specific agents coupled with chemotherapy, including the effects of pCR on disease-free survival and overall survival [43,44]. Lapatinib is another drug that acts on HER2 as an epidermal growth factor receptor (EGFR) and inhibits tyrosine kinase. Combined with capecitabine, lapatinib is administered in HER2-positive patients with advanced breast cancer [45,46].

Non-luminal tumors are characterized by non-expressing hormonal receptors and may express HER2 [28,47]. They are more common in young women and have a worse prognosis despite an initial good response to chemotherapy. The triple-negative breast cancer (TNBC) subtype is an undifferentiated carcinoma that is biologically aggressive and is usually detected in its advanced stages. Although TNBC presents with high rates of pCR after neoadjuvant chemotherapy with anthracycline and taxanes, a high rate of recurrence is observed among patients [47]. Preclinical and clinical studies suggest that women harboring TNBC may benefit from platinum-based chemotherapy. Randomized trials of patients with initial or advanced TNBC showed that platinum-based chemotherapy was generally associated with long-term survival [48,49,50]. Lapatinib may also be indicated as a treatment for TNBC because of its selective EGFR targeting. Additionally, it has clinical benefits regarding metastatic progression [46].

HER2-positive/ER- and PR-negative tumors are aggressive high-grade cancers that are usually self-detected and often observed in younger women [51]. Target therapies with anti-HER2 (trastuzumab), anti-HER2/HER3 (pertuzumab), or anti-HER2 and EGFR (lapatinib) can be used in these patients [36].

Although the classification of breast carcinomas into molecular or histological subtypes has contributed to a better stratification of patients into different therapeutic techniques, breast cancer nevertheless progresses or recurs in many women despite systemic therapy. Therefore, drug resistance remains a critical unsolved problem [51].

## 3. Drug Resistance in Breast Cancer

Drug resistance is the main factor responsible for cancer-associated deaths, and brings significant impairment to therapeutic interventions. Indeed, chemotherapy, the most common systemic treatment of breast cancer, benefits only 50% of users because of the development of resistance to multiple drugs [52]. For example, more than 30% of women with metastatic breast cancer do not respond to first-line chemotherapy based on anthracyclics and taxanes, and their disease typically progresses in less than 1 year [9]. Moreover, up to 50% of women with luminal carcinomas treated with endocrine therapy develop hormonal resistance. However, ER-regulatory pathways that could contribute to a hormone-resistant phenotype are still poorly understood [53].

Drug resistance may be inherent in first-line chemotherapy or hormone therapy, or the patient may develop resistance leading to disease progression some years after the initial treatment [9]. Resistance observed prior to treatment is innate (also known as intrinsic or de novo) and depends on the cancer subtype and a variety of factors influencing the tumor microenvironment [54]. Acquired resistance occurs through the growth of resistant cell clones, the type of drug used, or an accumulation of mutations in initial sensitive cells. Acquired resistance can be ascribed to pharmacological mechanisms, increased or decreased activity or gene expression, or changes in target molecules and other mechanisms [4].

Chemoresistance can be acquired through different molecular changes including epigenetic modifications [55], the inhibition of DNA repair proteins [56], the deregulation of proliferative and apoptotic pathways, metabolic alterations [57], an increase in autophagy [58], or the overexpression of adenosine triphosphate (ATP)-binding cassette (ABC) [59] efflux transporter or breast cancer resistance protein, which decreases intracellular drug concentrations. Breast cancer resistance protein is encoded by the *ABCG2* gene [60] and was shown to interact with other proteins responsible for drug transport mechanisms and chemoresistance [61]. Moreover, the interactions between tumor cells and their surrounding stroma may affect tumor behavior and contribute to therapeutic responses [62]. Therefore, tumor microenvironment pathway changes are also critical to treatment success. The deregulation of chemokines and cytokines in therapy, for instance, leads to the selection of tumor cell clones associated with chemoresistance [63]. Macrophages recruited after anti-cancer drug administration can protect tumor cells from death and induce chemoresistance [64]. Breast tumors have an accumulation of cancer-associated fibroblasts (CAFs), which are thought to promote chemoresistance [65]. Increasing evidence shows that CAFs interact with breast cancer cells, resulting in diverse responses to anti-cancer drugs, mostly through metabolic regulation or signaling pathway activation [66,67,68].

**Figure 1:**
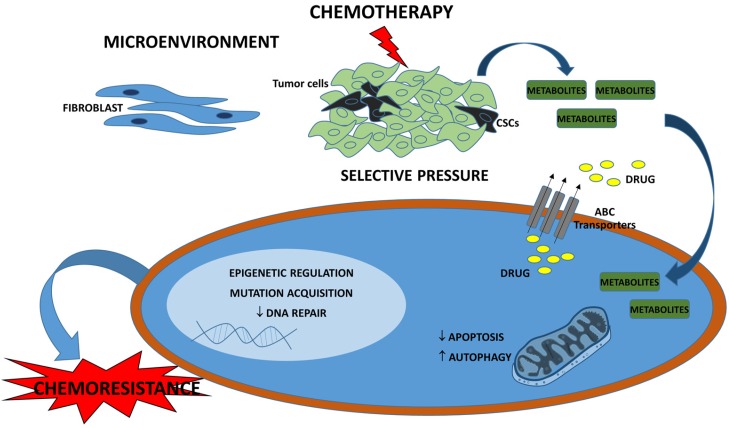
Chemotherapy agent could promote selective pressure of cancer stem cells (CSCs) and resistant cell clones and might increases the probability of recurrence. It may occur through pharmacological mechanisms, epigenetic modification, inhibition of DNA repair proteins, deregulation of proliferation and apoptotic pathways, metabolic alterations, autophagy increase, adenosine triphosphate (ATP)-binding cassette (ABC) efflux transporters overexpression that decreases the drug intracellular concentration. Moreover, the interactions between tumor cells and its surrounding microenvironment enriched by fibroblasts may also contribute to response to therapy.

The presence of the specific sub-population of cells, the cancer stem cells (CSCs), is another factor relevant to chemoresistance. CSCs are characterized by a self-renewing capacity, cell-surface marker CD44^+^/CD24^−/low^ expression, an enhanced capacity for tumor generation, and resistance to treatment because of their quiescent behavior [69]. Some studies have shown that TNBCs exhibit an enriched CSC population, which may favor tumor recurrence [70,71]. Accordingly, several reports recently demonstrated that breast cancer patients treated with neoadjuvant chemotherapy had an enrichment of CSCs and aggressive properties, which affect patient curability [72,73]. These factors together constitute important mechanisms to explain the high rate of breast cancer recurrence through acquired chemoresistance (Figure 1).

## 4. Current Metabolomic Approaches

MS and NMR are the main analytical tools employed in metabolome analyses. Biochemical data obtained and interpreted using these approaches provide a broader perspective of pathological processes than can be obtained from isolated biological markers. Metabolomics contributes to the diagnosis or treatment response of breast cancer by interpreting molecular measures using specific computational models to produce a clinically relevant result [70,74,75].

Metabolomics essentially uses targeted and untargeted approaches. Targeted metabolomics aims to identify a pathway or a metabolite of interest, based on a previously known relationship with a particular pathway or metabolite in the metabolome composition of an investigated sample. The untargeted approach seeks to identify and quantify the largest number of metabolites in a sample. Among the main techniques used in metabolomics studies, MS can be coupled to separation techniques such as liquid chromatography (LC-MS) or gas chromatography (GC-MS), as well as to NMR. Although NMR is a conservative technique and less sensitive than MS, its key advantages are that it is highly reproducible, quantitative, has a relatively low cost, and provides structural information for the accurate identification of metabolites [76]. Additionally, NMR does not use ionizing radiation, or require physical or chemical treatments prior to analysis, thus avoiding metabolite loss. Therefore, NMR is particularly useful in applications involving sensitive samples or living organisms [77].

## 5. Metabolic Profile of Breast Cancer

Cancer development occurs when different factors contribute to clonal evolution. These factors can be grouped into two major categories: the activation of oncogenes (e.g., MYC proto-oncogene (*MYC*), RAS type GTPase family (*RAS*), and/or phosphatidylinositol 3-kinase (PI3K-AKT-mTOR) pathways) that stimulate cell proliferation, and the inactivation of tumor suppressor genes involved in growth suppression (e.g., retinoblastoma-associated (*RB*) and tumor protein p53 (*TP53*)), DNA repair (breast cancer type 1/2 (*BRCA1*/*2*)), or proliferation-restrictive signaling (phosphatase and tensin homolog (*PTEN*)) [78,79]. When these changes are present in early stage cells, the affected individual has a high chance of developing cancer. However, in addition to these genetic alterations, the metabolic reprogramming of cells and adjacent stroma is required for cancer development. The current biological model of carcinogenesis and drug resistance considers various pathways, such as cell proliferation, evasion of the mechanisms involved in suppression of cell growth, resistance to cell death, genomic instability and mutations, replication of immortalized cells, induction of angiogenesis invasion and metastasis capability, tumor-induced inflammation, and evasion of the immune system [79,80].

Cancer and metabolism are deeply interconnected. Changes in metabolic networks, such as those involved in biosynthetic pathways, can greatly affect the metabolism of cancer cells [81]. Processes such as tumor development, tissue remodeling, cell survival changes, and metastasis are responsible for triggering these metabolic changes. Studies indicate that metabolism determines cancer evolution, and is allied with the action of a particular drug. In other words, metabolic adaptation is influenced by tumor microorganization [82]. The production of metabolites changes when tumor cells show altered metabolism, which results in a signature capable of characterizing the presence or even the behavior of the cancer. The metabolomic profile can also be altered by the surrounding stroma and immune response, providing complementary information about the tumor development and treatment response [83].

The metabolic profile of breast cancer cells differs from that of normal breast epithelial cells, and the metabolic profile of drug-sensitive breast cancer cells differs from resistant ones. Therefore, the analysis of metabolic pathways enables a better understanding of changes in metabolism that could promote carcinogenesis [22]. Normal human cells use glucose as a source of energy in the presence of oxygen. The glucose metabolized in the cytosol results in the production of pyruvate that enters mitochondria, is oxidized by the Krebs cycle, and culminates in the generation of ATP, the main source of cellular energy storage. However, even in aerobic conditions, most of the pyruvate in cancer cells is directed away from mitochondria and, under the action of lactic dehydrogenase, results in lactate. This process is typically observed in low oxygen environments. Lactate production in the presence of oxygen is known as aerobic glycolysis or the “Warburg effect” [78,84,85,86].

Breast cancer cells have an increased absorption of glucose [78], which is associated with activated oncogenes (*RAS* and *MYC*) and mutant tumor suppressors (*TP53*). These both interfere with proliferation, the inactivation of growth suppression, and the decrease of apoptosis. During neoplastic growth, progressive hypoxia occurs because of inefficient neovascularization leading to the expression of multiple enzymes involved in the glycolytic pathway [79]. As well as providing energy and biomolecules to cancer cells, glycolytic deviation contributes to cell–cell communication, thus reinforcing the hypothesis that a symbiosis known as the tumor microenvironment exists between cancer cells and adjacent stroma. In cancer, lactate acts as a source of energy and molecular signaling, mimicking physiological mechanisms of high anaerobic performance. The complexity of a tumor microenvironment and the interconnections between different cell types make it difficult to understand the lactate circuit [87].

Recent research aimed to identify metabolic pathway changes associated with breast carcinogenesis. Using a large-scale methodology, Jain et al. [88] recognized that the glycine biosynthetic pathway was highly correlated with fast proliferating breast cancer cells. They suggested that glycine consumption is required for cancer cell proliferation, and is associated with worse prognosis in breast cancer patients. Their findings also suggested a potential cancer biomarker and therapeutic response tracking [88].

In an in vitro analysis, Xie et al. [89] reported that aspartate levels were higher in the MCF-7 cell line than in MCF-10A cells. The low levels of aspartate found in the blood of breast cancer patients suggested that amino acids were being consumed as part of tumor development. These results indicated that circulating aspartate is a key metabolite characteristic of human breast cancer [89]. Another in vitro analysis of MCF-7 and MDA-MB-231 cells used NMR to identify metabolites and quantify inositol 1,4,5-trisphosphate receptors (IP3R). This revealed the functional relevance of IP3R in causing metabolic disorders, resulting in reduced glucose uptake in both cell lines. Metabolomic analysis was also used to study changes in breast cancer metabolism with an emphasis on glutamine and its transporters. Glutamine is considered one of the main amino acids involved in tumor development. The authors used in vivo analysis to identify serum metabolites in breast cancer patient, which showed that IP3R expression was up-regulated in many cases. An increase in lipoprotein content and levels of metabolites such as lactate, lysine, and alanine, and a decrease in serum pyruvate and glucose levels, were also observed in patients who presented with high IP3R levels compared with healthy individuals [90].

In an analysis of serum from breast cancer patients and healthy controls, GC-MS was used to obtain metabolic profiles, followed by chemometric analysis to differentiate which metabolites showed substantial changes. Pathway analysis revealed metabolic alterations in breast cancer patients evidencing increased glycolysis, lipogenesis, and the production of volatile organic metabolites compared with healthy women [91]. Also comparing the metabolic profile of serum samples from healthy women with subtype-independent breast cancer patients, Jové et al. [92] identified 1269 metabolites with different serum concentrations in both groups and 354 metabolites belonging to aminoacyl-tRNA biosynthesis, arginine and proline metabolism, and primary bile acid biosynthesis pathways. Caproic acid and stearamide were identified as metabolites significantly associated with disease. Patients with early stage cancer had increased serum levels of choline, tyrosine, valine, lactate, isoleucine, and decreased glutamate levels. However, in women with metastatic cancer, serum glucose and glutamine levels were shown to decrease. The authors argued that differences in oncogene expression are correlated with the metabolic profile, which may lead to disease relapse [92]. In another study, serum lipid concentrations were evaluated in women with newly diagnosed invasive breast cancer at stages I and II. NMR was used for the metabolomic analysis of serum lipoprotein subfractions, which revealed an association between lipoproteins and ER expression. However, an inverse association between subfractions of high density lipoprotein and Ki67 was noted, and low density lipoproteins were positively associated with nodal metastasis. Therefore, it was possible to associate subfractions of lipoproteins with a characteristic of breast cancer acting on the aggressiveness and prognosis of the tumor. These results suggested an association between different lipoprotein subfractions and the expression of PR and Ki67 in breast tumors [93]. 

Through the metabolomic analysis of serum and plasma samples from two groups of patients with primary breast cancer, Xie et al. showed that breast cancer was associated with low plasm levels of aspartate due to higher levels of aspartate in breast cancer tissues in consequence of increased tumor aspartate utilization [89]. Evaluating the plasma metabolism of patients with early or metastatic breast cancer by NMR, they also observed variations in glucose, lactate, pyruvate, alanine, leucine, isoleucine, glutamate, glutamine, lysine, glycine, threonine, tyrosine, phenylalanine, acetate, acetoacetate, β-hydroxybutyrate, urea, creatine, and creatinine. In particular, lactate levels were inversely correlated with tumor size in the cohort of patients with early breast cancer. It has been suggested that tumor cells are capable of inducing modulation of the patient’s metabolism even in early stages of the disease [94].

Fuss et al. [95] emphasized the importance of evaluating a complete metabolomic profile rather than correlating isolated metabolites because of its greater ability to predict prognosis. They analyzed the role of cancer metabolism using ex vivo high-resolution magic angle spinning (HR-MAS) to study the metabolic profiles of intact breast tissue. Compared with benign tissue, levels of compounds containing taurine and choline were elevated in breast tissue. Patients reported to be healthy up to five years after surgery were found to have increased levels of taurine, glycerophosphocholine, and creatine, with decreased levels of glycine and phosphocholine in their malignant tissues [95]. In an analysis of primary tumor samples from un-treated breast cancer patients, the authors used HR-MAS magnetic resonance spectroscopy (MRS) to identify three significant metabolic clusters: one had the highest levels of glycerophosphocholine and phosphocholine, the second had the highest levels of glucose, and the third had the highest levels of lactate and alanine. Interestingly, the genetic subtypes were uniformly found among the three metabolic clusters. The metabolic clusters could contribute to explaining the heterogeneity of breast cancer [96].

Ansari et al. [97] concluded that understanding the metabolic pathways of different breast cancer subtypes may lead to the discovery of potential biomarkers to help in the orientation of personalized treatments. Discrepancies among molecular classes of breast cancer are apparent for some metabolic pathways, such as the glutamine pathway in TNBC, which has an aggressive metabolic pattern. Although previous studies have undoubtedly shown the usefulness of the metabolomics approach, the establishment of future validation using independent cohorts is essential to understanding the relevance of specific metabolic biomarkers [97].

## 6. Metabolomic-Based Breast Cancer Chemoresistance

Recently, several in vitro, ex vivo, and in vivo studies have been performed to understand the metabolic pathways involved in breast cancer drug resistance (Table 1). Among the major in vitro studies, Ryu et al. [98] observed that glycolysis, as well as the production of lactates and ATP, is associated with resistance to adriamycin in MCF-7 cells. Their results suggest that the regulation of sulfur amino acid metabolism may be a therapeutic target for chemoresistant cells [98]. Using the same cell line, Cao et al. [99] observed that adriamycin deaccelerated several metabolic pathways, including purine, pyrimidine, glutathione, and glycolysis routes, as well as aggravating oxidative stress. These findings suggest that cellular metabolomics and the quantitative measurement of metabolic markers can be used to evaluate antitumor effects and investigate antitumor candidate agents [99]. In MCF-7 cells exposed to ascididemine, Morvan [100] observed an increase in citrate, gluconate, and polyunsaturated fatty acids, and a decrease in glycerophosphocholine and ethanolamine associated with severe oxidative stress in vitro. He concluded that central metabolic changes in breast cancer cells are responses to high oxidative stress [100]. Similarly, Bayet-Robert and Morvan [101] reported changes in glutathione and lipid metabolism as well as glucose use in MCF-7 and MDA-MB-231 cells exposed to curcumin and docetaxel [100,101].

Comparing metabolic pathways in luminal A breast cancer cells (BT474 and MCF-7) and triple-negative cells (MDA-MB-231 and MDA-MB-468), Stewart et al. [102] observed different metabolic responses to paclitaxel treatment. For example, in both luminal A and triple-negative cells, choline and its metabolites increased in the presence of paclitaxel. Moreover, choline, acetylcholine, phosphocholine, and sn-glycero-3-phosphocholine increased under treatment in MDA-MB-468 but not MDA-MB-231 cells, except for sn-glycero-3-phosphocholine. The myo-inositol level also increased during treatment and was higher in luminal A cells compared with triple-negative cells. Based on these studies, it was notable that glycolysis and glutathione pathways were deregulated when cells were treated with adriamycin and docetaxel. This suggested that new studies should focus on these biochemical pathways to expand our understanding of chemotherapeutic effects as well as possible mechanisms of resistance [102]. 

Using human serum samples, Miolo et al. [103] investigated biomarkers potentially associated with pCR in the treatment of neoadjuvant trastuzumab-paclitaxel in HER2-positive breast cancer patients through a pharmacometabolomics approach. Serum levels of spermidine and tryptophan identified patients who achieved pCR with a high sensitivity. These results were useful for elucidating individual metabolic responses to treatment, and may help select the most suitable patients for treatment with trastuzumab-paclitaxel [103].

Using HR-MAS NMR spectroscopy technology, Maria et al. [104] studied the in vitro metabolic profile of human breast cancer cells treated with tamoxifen, cisplatin, and doxorubicin. The study findings emphasized that different breast tumor lines respond in remarkably different ways to chemotherapy. It was also observed that changes in acetate, lactate, and phosphocholine helped identify tumor response to a given treatment based only on molecular properties [104].

Wei et al. [105] investigated the toxicity mechanism of 2,2′,4,4′-tetra-bromodiphenyl ether (BDE-47) in MCF-7 breast cancer cells. Metabolomic analysis using ultra-high performance LC-MS showed that toxicity to MCF-7 cells increased gradually when the concentration of BDE-47 exceeded 1 mM. BDE-47 was found to induce oxidative stress by inhibiting pathways involving pyrimidine and purine, and the pentose phosphate pathway (PPP), and disrupting the entire cell metabolism. Thus, pyrimidine and purine metabolism could be reduced by downregulating mRNA transcripts, and oxidative stress could be induced by inhibiting nicotinamide adenine dinucleotide phosphate hydrogen (NADPH) in the PPP observed in MCF-7 cells exposed to BDE-47 [105]. 

Based on an ex vivo model, van Asten et al. [106] observed that breast cancer tissues of syngeneic mice (K14cre; Brca1^F/F^p53^F/F^) resistant and sensitive to docetaxel showed different modifications of metabolic pathways during treatment. Evaluating the tumors sensitive to docetaxel, the authors observed that the metabolic profile 48 h after drug treatment was characterized by a high level of phosphocholine compared with untreated tumors. Within the first 48 h of treating sensitive tumors, the observed proportion of total choline, glycerophosphocholine, phosphocholine, and creatinine was significantly increased. They concluded that docetaxel-sensitive tumors have an increase of metabolites containing choline, as observed 1–2 days after beginning therapy, which corresponded with the time of higher apoptotic activity. In docetaxel-resistant tumors, the metabolites derived from choline did not increase during treatment. However, relative concentrations of choline components were higher in the pre-treatment of docetaxel-resistant tumors than in sensitive tumors [106].

Euceda et al. [107] used HR-MAS MRS to analyze human breast tumor samples. The tumors were biopsied before, during, and after neoadjuvant chemotherapy. Metabolites of all observed constituents of total choline significantly decreased post-treatment, and were significantly lower in sensitive patients compared with a resistant patient. A significantly lower level of succinate was also observed in sensitive patients. Unexpectedly, the authors found a significant increase in lactate with treatment progression in sensitive patients. Both an increase in lactate production and rapid glucose consumption are characteristic of the Warburg effect. They also observed changes in glutathione metabolism identified as a possible effect of bevacizumab [107].

Few studies have evaluated serum metabolomic changes in women with breast cancer. Wei et al. [108] compared the serum metabolic profile of HER2-positive women with a pCR, a partial response, and with stationary disease following neoadjuvant chemotherapy with epirubicin and cyclophosphamide followed by doxorubicin associated with trastuzumab. They identified a progressive increase in threonine, glutamine, and linoleic acid in patients with a pCR, followed by those with a partial response and stationary disease with the progressive reduction of isoleucine. The underlying mechanism of this distinction in resistant and sensitive patients is not fully understood. In vivo analyses showed that the linoleic acid pathway was the most affected after doxorubicin treatment [108].

## 7. Future Perspectives

Metabolomic analytical techniques are distinguished by the level of sensitivity, volume of material to be analyzed, and sample preparation methods. Analytical platform improvements have allowed the high-throughput collection of different molecular levels with large amounts of data. These multi-layer data omics enable a clearer view of biological systems to be obtained because they do not only focus on single-layer omics. Given the complementary nature of different molecular levels, multi-layer data omics facilitate understanding and applicability in clinical routine. Metabolomics is therefore an attractive approach for providing information about cancer biology because it is obtained through a metabolic profile and is associated with complementary methods [96].

Recent studies have focused on in vitro and in vivo approaches. However, few have correlated both approaches to validate the methodology. Additionally, few have evaluated the different subtypes of breast cancer with respect to functions of time, stage, drugs, and duration of treatment. Studies in clinical cohorts should therefore be performed to recognize the potential of data to predict results and follow up on breast cancer treatment. It is also important that specialized oncologists work with other health professionals to improve the analysis of results obtained from methodological tools and present them in a format that is helpful for managing routine patients. Use of the metabolomic approach in clinical routine helps decipher the main regulatory pathways in different breast cancer subtypes. The clarification of individual behavioral changes in both disease development and treatment response is essential for developing more effective treatments and customizing cancer treatments [109].

Breast cancer is a heterogeneous disease, and chemotherapy failures are caused by drug resistance, which is a leading cause of breast cancer mortality. The metabolic analysis of fluids and tissues of cancer patients contributes to an understanding of the metabolic pathway reprogramming involved in neoplastic transformation, prognosis, and drug resistance [78,79]. Several studies have been proposed to evaluate metabolic pathway reprogramming in chemoresistance, and identify patients who are resistant to chemotherapy. However, studies that verify whether metabolic pathways are associated with the response to chemotherapy are lacking. Such studies could provide evidence for use in clinical practice, while the identification of different metabolic profiles may suggest new molecular targets and metabolic biomarkers that will contribute to patient stratification of different breast cancer subtypes. Finally, the knowledge of specific metabolic pathways could impact on the evaluation of new drugs with possible repercussions on the survival of breast cancer patients. The prompt identification of chemotherapy-resistant tumors would aid with earlier and more accurate stratification of patients, and the choice of adjusted therapeutic regimens [74,105].

## Figures and Tables

**Table 1:** Studies involving metabolic pathway changes based on different treatments.

Biological Materials	Approach	Specific Treatment	Metabolic Pathways Identified	Reference
MCF-7	Immunoblot analyses	Adriamycin	Sulfur amino acid metabolism	[98]
MCF-7	GC-MS	Adriamycin	Increase in glycerol metabolism and decrease in glutathione biosynthesis.	[99]
MCF-7	NMR	Ascididemin	Increase in citrate, gluconate and polyunsaturated fatty acids and decrease in glycerophospho-choline and ethanolamine.	[100]
MCF-7 MDA-MB-231	NMR	curcumin +/− docetaxel (dose- and time-response)	Changes in glutathione metabolism, lipid metabolism, and glucose utilization—some biphasic changes depending on exposure.	[101]
BT474 MCF-7 MDA-MB-231 MDA-MB-468	NMR	Paclitaxel	In luminal A cell lines: lactate and creatine decreased while certain choline metabolites and myo-inositol increased with paclitaxel. In TNBC cell lines: glutamine, glutamate, and glutathione increased, whereas lysine, proline, and valine decreased in the presence of drug.	[102]
Human serum samples	LC-MS	Trastuzumab-placlitaxel	Changes in spermidine and tryptophan.	[103]
MDA-MB-231	HR-MAS NMR	Tamoxifen, cisplatin and doxorubicin	Changes in acetate, lactate and phosphocholine.	[104]
MCF-7	UHPLC-MS	Polybrominated diphenyl ethers (PBDEs)	Change in the pentose phosphate pathway.	[105]
Tissue samples mouse model	HR-MAS	Docetaxel	In docetaxel-sensitive tumors: increase in choline metabolites. In tumors resistant to docetaxel: metabolites derived from choline did not increase during treatment.	[106]
Human breast tumor tissue	HR-MAS	5-Fluorouracil, epirubicin, cyclophosphamide followed by taxane randomized to bevacizumab	Lower glucose and higher lactate was observed in patients exhibiting a good response compared to those with no response	[107]
Human serum samples	LC-MS NMR	Epirubucin and cyclophosphamide followed of doxorubicin in association to trastuzumab in HER2-positive cases	Concentrations significantly different threonine, isoleucine, glutamine and linolenic acid.	[108]
